# Dietary carotenoids intake and sex differences in relation to chronic kidney disease a cross-sectional assessment in the NHANES study

**DOI:** 10.1186/s12889-024-17771-z

**Published:** 2024-01-24

**Authors:** Yumeng Shi, Yanjie Xu, Wei Zhou

**Affiliations:** 1 Department of Cardiovascular Medicine, The Second Affiliated Hospital, Jiangxi Medical College, Nanchang of Jiangxi, Nanchang University, No. 1 Minde Road, 330006 Jiangxi, China; 2 Center for Prevention and Treatment of Cardiovascular Diseases, the Second Affiliated Hospital, Jiangxi M edical College, Nanchang of Jiangxi, Nanchang University, Nanchang, China; 3 Jiangxi Provincial Cardiovascular Disease Clinical Medical Research Center, Nanchang of Jiangxi, Jiangxi, China; 4 Jiangxi Sub-center of National Clinical Research Center for Cardiovascular Diseases, Jiangxi, China

**Keywords:** Dietary carotenoids, Chronic kidney disease

## Abstract

**Background and aims:**

Current evidence on the relationship between dietary carotenoids intake (DCI) and chronic kidney disease (CKD) are limited. Moreover, whether there is an association between DCI and prevalence of CKD and how this association might be impacted by sex is not clear.

**Methods and results:**

Overall, 4507 women and 4396 men were included for analysis. The study used data from the National Health and Nutrition Examination Survey (NHANES), a cross-sectional survey carried out in the USA. The exposure factor for this analysis was DCI. The outcome was CKD, defined as eGFR < 60mL/min/1.73 m^2^. Multivariate logistic regression was used to analyzed the associations of DCI and prevalence of CKD both in men and women. There was a significant inverse association between higher DCI and prevalence of CKD only among females. Per LgDCI unit increment, the multivariable adjusted odd ratio (OR) (95% confidence interval) for prevalence of CKD was 0.72 (0.58, 0.90) in females and 0.95 (0.77, 1.18) in males. When compared with the lowest quartile, the ORs of the highest quartile of DCI for prevalence of CKD were 0.59 (0.40, 0.87) in females and 0.87 (0.60, 1.26) in males. The associations remained similar in the subgroup analyses.

**Conclusions:**

The findings suggest that a higher intake of dietary carotenoids is associated with a lower prevalence of CKD in women, but not in men.

## Highlights

The present study provides evidence that a higher DCI is specifically associated with a reduced prevalence of CKD in women, regardless of other potential confounding factors for CKD.

In clinical practice, female patients can be encouraged to eat more foods rich in carotenoids to prevent the occurrence and development of CKD and related cardiovascular diseases, while for men, it is still the main risk factor for the control of CKD.

## Introduction

Chronic kidney disease (CKD) presents a global public health challenge, as indicated by the data from the Global Burden of Disease Research. In 2017, an estimated 697 million individuals worldwide were affected by CKD, reflecting an average prevalence rate of 9.1% [1]. CKD is associated with an increased risk of death from cardiovascular diseases (CVD), and significantly increases the CVD risk of patients with hypertension and diabetes [2–4]. Despite the control of related risk factors such as hypertension and diabetes, the burden of CKD has not been alleviated in many parts of the world. This may be due to low awareness of the disease among both the public and health-care authorities of the importance of prevention at the primary level through the addition of relevant healthy eating patterns such as fresh fruits and vegetables. There is evidence that higher intake of fruits and vegetables can reduce the risk of CKD [5–7].

By combining with the pathological process of CKD, we know that it is a chronic inflammatory disease involving oxidative stress, which is a key step in its occurrence and progression [8]. Therefore, to prevent the occurrence and progression of CKD, it is imperative to address the underlying pathological mechanisms. Supplementation of natural dietary antioxidants can effectively mitigate oxidative stress and inflammatory reactions in the human body. Carotenoids are strong antioxidants and are widely present in fruits, vegetable, fish and other foods [9]. These carotenoids include beta-carotene, alpha-carotene, lycopene, lutein, and beta-cryptoxanthin in the diet and human body [10], which all have unique antioxidant properties [11]. Besides, epidemiological studies have shown a correlation between high dietary carotenoids and a reduced risk of breast cancer [12], cervical cancer [13], ovarian cancer [14], colorectal cancer [15], and CVD [16, 17]. However, no previous study explored the correlation between dietary carotenoid intake (DCI) and CKD, and only one study evaluated the relationship between serum carotenoids and CKD. The results show that there is a significant correlation between the increase in serum carotenoids and the decrease in CKD [18]. A more comprehensive evidence base on diet and the risk of CKD will improve dietary recommendations and provide more stringent guidelines for dietary interventions in populations at high risk for CKD. Due to a higher prevalence of smoking among men compared to women, the intake of nicotine and subsequent oxidative stress reactions during smoking can inhibit and reduce levels of carotenoids in the body [19]. Consequently, even if men consume more dietary carotenoids, its protective action may be compromised.

Therefore, the present study aims to investigate the impact of dietary carotenoids intake on CKD in both male and female populations, respectively. It will be verified by cross-sectional data from the National Health and Nutrition Examination Survey (NHANES) database.

## Methods

### Study design and population

Data for this study were obtained from the NHANES database, and all data were available free of charge on the NHANES official website https://www.cdc.gov/nchs/nhanes/. NHANES database is a series of representative large multi-stage sampling survey projects in the United States [20]. The database is designed to collect information about the health and nutrition of the U.S. population through interviews, physical exams, and laboratory tests. A detailed survey operating manual, consent documents, and pamphlets for each period are available on the NHANES website https://www.cdc.gov/nchs/nhanes/index.htm. All study protocols were reviewed and approved by the National Center for Health Statistics (NCHS) Ethics Committee, and data were collected with the written informed consent of the participants. This cross-sectional analysis comprised 19,530 participants aged over 18 years who were enrolled in the NHANES between 2011 and 2018, all of whom underwent renal function testing and completed a dietary questionnaire. We excluded individuals with missing covariate values (see the Potential Covariates section; *N* = 10,627). Finally, 8,903 subjects were included in our study.

### Definition of the dietary carotenoids intake and CKD

The exposure factor for this analysis was DCI, the mean dietary intake derived from two 24-hour recall surveys conducted by trained interviewers.The dietary carotene intake was assessed using a semi-quantitative food frequency questionnaire, comprising 131 different types of foods and inquiries regarding vitamin and mineral supplements. Participants were asked to report the average consumption frequency of a specific unit or portion size for each food item over the past year (e.g., an apple or a slice of bread). Response options ranged from “never” to “≥6 times/day”. The intake score was calculated by multiplying the nutritional content of each food item by its reported frequency of consumption, utilizing data on food composition from sources such as the US Department of Agriculture (USDA), food manufacturers, and other publicly available resources [21–23]. The carotenoid food ingredient database comprises the prevalent carotenoids found in fruits and vegetables, namely alpha-carotene, beta-carotene, lutein and zeaxanthin, beta-cryptoxanthin and lycopene. The tomato-based foods’ carotenoid content has been updated based on the USDA database [24]. The outcome was CKD, defined as eGFR < 60mL/min/1.73 m^2^ [25]. The eGFR in this study was calculated using the Chronic Kidney Disease Epidemiology Collaboration (CKD-EPI) formula [26]. This formula estimates GFR based on serum creatinine levels, incorporating factors such as age, sex, and race.

### Potential covariates

NHANES provided information about age, sex, race, body mass index (BMI), smoking status (never, former, or current), systolic blood pressure (SBP), diastolic blood pressure (DBP), fasting plasma glucose (FPG), total cholesterol (TC), high-density lipoprotein cholesterol (HDL-C), serum uric acid (SUA), antihypertensive drugs, lipoprotein-lowering drugs, and glucose-lowering drugs. Hypertension was defined as self-reported physician diagnosis of hypertension, or a SBP ≥ 140 mmHg and/or DBP ≥ 90 mmHg, or using antihypertensive drugs. Diabetes was defined as self-reported physician diagnosis of diabetes or FPG concentration ≥ 7.0 mmol/L or use of glucose-lowering drugs.

### Statistical analysis

Since DCI was skewed and distributed leftward, we performed a log10 conversion when it was a continuous variable. Quantile Quantileplot (Q-Q plot) and the Anderson‒Darling test [27] were used to test the normality of the evaluation variables in this study. Baseline characteristics of men and women are presented according to quartiles of DCI. Continuous variables are shown as mean ± standard deviation (SD), and categorical variables are shown as n (%) for categorical variables. Descriptive analyses were conducted according to DCI quartiles using One-way analysis of variance and the chi-square test to compare between-group differences. Multivariate logistic regression was used to determine odd ratios (OR) and 95% confidence intervals (95%CI) for the associations of DCI and prevalence of CKD both in man and woman. Covariates were included as potential confounders in the final multivariate logistic regression models if their inclusion resulted in a change of more than 10% [28] in the estimates of DCI on CKD, or if they were recognized as traditional risk factors for CKD.We established two models: Model 1 was adjusted for age, race, BMI, smoke, FPG, TC, HDL, SUA, and model 2 was adjusted for age, race, BMI, smoke, FPG, TC, HDL, SUA, lipoprotein-lowering drugs, glucose-lowering drugs, diabetes, hypertension. In addition, a generalized additive model and fitting smooth curve (penalty spline method) were used to further describe the dose‒response association between DCI and CKD among men and women. Stratification analyses according to age (< 65 vs. ≥ 65 years), race (non-Hispanic white vs. non-Hispanic Black vs. Mexican American vs. other Hispanic vs. other races), BMI (< 24 vs. ≥24 kg/m^2^), smoking status (never vs. former vs. current), diabetes (no vs. yes), and hypertension (no vs. yes) were performed to test whether these factors could modify the association between DCI and CKD intervals men and women.

All data analyzed were using the statistical package R (http://www.r-project.org) and Empower (R) (www.empowerstats.com). A 2-tailed *P* < 0.05 was considered to be statistically significant.

## Results

### Study participants and baseline characteristics

A total of 4507 women were included in the study, with the average age (standard deviation: SD ) of 48.8 (18.0)years old, and the dietary carotenoids intake (DCI) of median and interquartile range (IQR) was 4872 µg (IQR: 1827.00-11286.00). The prevalence of hypertension and diabetes were 1902 (42.2%) and 782 (17.3%) respectively. A total of 4396 men, with an average age (SD) of 48.7(18.2) years, median dietary carrotene intake was 5438 µg (IQR: 2031–12,681), of which 1892 (43.0%) had hypertension and 879 (20%) had diabetes. Table 1 compares the characteristics of the study participants which are divided into four quartiles by DCI among males. Compared with the lower quartiles, the upper quartiles group of DCI was more likely to be patients who were non-Hispanic whites and current smokers. However, there were no statistical differences among the four groups in age, BMI, SBP, DBP, FPG, TC, HDL-C, SUA, diabetes, hypertension or medication history (*p* > 0.05). Table 2 shows the characteristics of the female participants. There were no marked differences in baseline characteristics for age, DBP, FPG, TC, SUA, diabetes, hypertension, antihypertensive drugs, lipoprotein-lowering drugs or glucose-lowering drugs according to DCI quartiles (*p* > 0.05). Compared to participants in the lowest quartile of DCI, those in the highest quartile exhibit several distinguishing characteristics: non-smoking habits, lower BMI and SBP values, as well as higher HDL-C levels.

**Table 1:** Baseline characteristics of study participants according to DCI among males

Variable^a^	DCI Quartiles				***P*** value
	Q1 (< 2031)	Q2 (2031 to < 5438)	Q3 (5438 to < 12,684)	Q4 (≥ 12,684)	
Males, n	1097	1101	1099	1099	
Age, year	49.64 ± 18.78	48.87 ± 18.51	48.29 ± 18.18	48.12 ± 17.31	0.197
BMI, kg/m^2^	28.59 ± 6.23	28.58 ± 6.22	28.73 ± 6.30	28.56 ± 6.24	0.913
Race					< 0.001
Non-Hispanic White, N(%)	434 (39.56%)	463 (42.05%)	420 (38.22%)	448 (40.76%)	
Non-Hispanic Black, N(%)	289 (26.34%)	212 (19.26%)	179 (16.29%)	205 (18.65%)	
Mexican American, N(%)	131 (11.94%)	143 (12.99%)	194 (17.65%)	150 (13.65%)	
Other Hispanic, N(%)	103 (9.39%)	114 (10.35%)	125 (11.37%)	99 (9.01%)	
Other races, N(%)	140 (12.76%)	169 (15.35%)	181 (16.47%)	197 (17.93%)	
Current smoking, N(%)	316 (28.81%)	315 (28.61%)	345 (31.39%)	318 (28.94%)	0.008
SBP, mmHg	126.42 ± 17.91	124.91 ± 16.65	124.70 ± 16.41	125.03 ± 17.45	0.076
DBP, mmHg	70.90 ± 12.39	70.98 ± 11.64	71.36 ± 11.82	72.08 ± 11.99	0.084
FPG, mg/dL	112.02 ± 33.81	112.04 ± 35.40	114.53 ± 40.28	113.58 ± 41.13	0.320
TC, mg/dL	182.69 ± 42.67	184.64 ± 41.87	186.07 ± 40.63	185.55 ± 40.36	0.237
HDL-C, mg/dL	48.86 ± 14.20	49.16 ± 14.59	48.69 ± 13.48	49.05 ± 14.31	0.872
SUA, mg/dL	6.14 ± 1.33	6.08 ± 1.33	6.05 ± 1.31	6.04 ± 1.30	0.275
Diabetes^$^	223 (20.33%)	219 (19.89%)	218 (19.84%)	219 (19.93%)	0.991
hypertension	499 (45.49%)	454 (41.24%)	461 (41.95%)	478 (43.49%)	0.189
Antihypertensive drugs	44 (4.01%)	61 (5.54%)	71 (6.46%)	56 (5.10%)	0.274
Lipoprotein-lowering drugs	248 (22.61%)	263 (23.89%)	250 (22.75%)	252 (22.93%)	0.980
Glucose-lowering drugs	146 (13.31%)	148 (13.44%)	151 (13.74%)	142 (12.92%)	0.997

^a^Data are presented as number (%) or mean ± standard deviation

Abbreviation: DCI: dietary carotenoids intakes; BMI: body mass index; SBP: systolic blood pressure; DBP: diastolic blood pressure; FPG: fasting plasma glucose; TC: total cholesterol; HDL-C: high-density lipoprotein cholesterol; SUA: serum uric acid

^$^diabetes was defined as self-reported physician diagnosis of diabetes or FPG concentration?7.0 mmol/L or use of glucose-lowering drugs.

**Table 2:** Baseline characteristics of study participants according to DCI among females

Variable^a^	DCI Quartiles				***P*** value
	Q1 (< 1827)	Q2 (1827 to < 4872)	Q3 (4872 to < 11,288)	Q4 (≥ 11,288)	
Females, n	1126	1127	1127	1127	
Age, years	49.06 ± 18.59	48.57 ± 18.07	49.02 ± 17.76	48.51 ± 17.63	0.834
BMI, kg/m^2^	30.40 ± 7.98	30.24 ± 7.94	29.79 ± 7.88	28.85 ± 7.58	< 0.001
Race					< 0.001
Non-Hispanic White, N(%)	442 (39.25%)	420 (37.27%)	440 (39.04%)	422 (37.44%)	
Non-Hispanic Black, N(%)	328 (29.13%)	262 (23.25%)	192 (17.04%)	221 (19.61%)	
Mexican American, N(%)	109 (9.68%)	160 (14.20%)	180 (15.97%)	169 (15.00%)	
Other Hispanic, N(%)	122 (10.83%)	136 (12.07%)	136 (12.07%)	101 (8.96%)	
Other races, N(%)	125 (11.10%)	149 (13.22%)	179 (15.88%)	214 (18.99%)	
Current smoking, N(%)	244 (21.67%)	173 (15.35%)	156 (13.84%)	146 (12.95%)	< 0.001
SBP, mmHg	124.25 ± 20.84	122.64 ± 19.53	121.79 ± 18.93	120.45 ± 18.66	< 0.001
DBP, mmHg	68.51 ± 12.06	68.66 ± 11.65	68.90 ± 10.96	68.93 ± 10.97	0.789
FPG, mg/dL	108.70 ± 37.34	108.76 ± 33.21	106.92 ± 32.02	106.29 ± 33.99	0.210
TC, mg/dL	190.56 ± 41.41	192.00 ± 42.17	192.33 ± 39.93	192.30 ± 40.32	0.700
HDL-C, mg/dL	57.07 ± 16.40	58.39 ± 16.03	58.57 ± 16.27	60.12 ± 17.06	< 0.001
SUA, mg/dL	4.99 ± 1.36	4.90 ± 1.33	4.89 ± 1.27	4.84 ± 1.24	0.061
Diabetes^$^	191 (16.96%)	216 (19.17%)	206 (18.28%)	169 (15.00%)	0.052
hypertension	506 (44.94%)	471 (41.79%)	469 (41.61%)	456 (40.46%)	0.165
Antihypertensive drugs	75 (6.66%)	67 (5.94%)	82 (7.28%)	69 (6.12%)	0.731
Lipoprotein-lowering drugs	239 (21.23%)	235 (20.85%)	215 (19.08%)	199 (17.66%)	0.351
Glucose-lowering drugs	125 (11.10%)	155 (13.75%)	153 (13.58%)	113 (10.03%)	0.081

^a^Data are presented as number (%) or mean ± standard deviation

Abbreviation: DCI: dietary carotenoids intake; BMI: body mass index; SBP: systolic blood pressure; DBP: diastolic blood pressure; FPG: fasting plasma glucose; TC: total cholesterol; HDL-C: high-density lipoprotein cholesterol; SUA: serum uric acid

^$^diabetes was defined as self-reported physician diagnosis of diabetes or FPG concentration ≥ 7.0 mmol/L or use of glucose-lowering drugs

### Dietary carotenoids intake and CKD

**Fig. 1:**
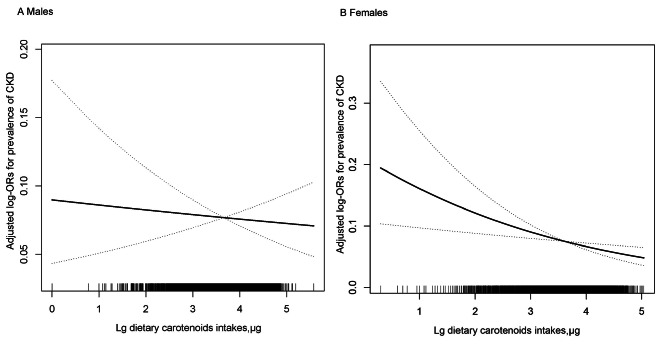
Association between DCI and the prevalence of CKD by sex. **(A)** males **(B)** females. A linear association between DCI and the prevalence of CKD was found (*P* < 0.05). The solid line and dashed line represent the estimated values and their corresponding 95% confidence interval. Adjustment factors included age, race, BMI, smoking, FPG, TC, HDL, SUA, lipoprotein-lowering, drugs, glucose-lowering drugs, diabetes, and hypertension

There was a significant inverse association between higher DCI and CKD among females (Fig. 1B).When adjustments were made using model 1 (age, race, BMI, smoke, FPG, TC, HDL, SUA), the prevalence of CKD in females was significantly lower with each incremental unit of LgDCI (OR: 0.74, 95%CI:0.59, 0.92, *P* = 0.007). Significant differences between DCI and CKD (OR: 0.72, 95%CI:0.58, 0.90, *P* = 0.004) remained in the fully adjusted model 2. As shown in Table 3 the ORs for women across the three upper quartiles were 0.67 (95% CI 0.46–0.97), 0.58 (0.40–0.86), and 0.59 (0.40–0.87), respectively, when compared with the lowest quartile (P for trend = 0.005). In men, by contrast, there was no correlation between DCI and the prevalence of CKD in men, using either model 1 or model 2. In model 2, compared with male participants with the lowest DCI, the multivariable adjusted OR for CKD was 0.65 (95% CI: 0.45, 0.95), 0.82 (95% CI: 0.57, 1.19) and 0.87 (95%CI:0.60, 1.26) for the other three groups. The odds ratios from the quartile analysis same not reach statistical significance (*P* for trend = 0.635). Figure 1A displays the dose response of DCI and CKD effects in male participants and shows similar results with the straight line gradually flattening out.

**Table 3:** Association of dietary carotenoids intakes with CKD among males and females^1^

DCI, ug	Events (%)	CKD OR (95%CI), ***P*** value	
		model 1	model 2
Male			
Continuous (Lg DCI)	338(7.64%)	0.96 (0.78, 1.19) 0.731	0.95 (0.77, 1.18) 0.669
Q1(< 2031)	107 (9.75%)	Reference	Reference
Q2(2031 to < 5438)	78 (7.08%)	0.66 (0.46, 0.96) 0.028	0.65 (0.45, 0.95) 0.025
Q3(5438 to < 12,684)	74 (6.73%)	0.84 (0.58, 1.21) 0.342	0.82 (0.57, 1.19) 0.303
Q4(≥ 12,684)	79 (7.19%)	0.89 (0.62, 1.29) 0.534	0.87 (0.60, 1.26) 0.465
*P for trend*		0.721	0.635
Female			
Continuous (Lg DCI)	338(7.50%)	0.74 (0.59, 0.92) 0.007	0.72 (0.58, 0.90) 0.004
Q1(< 1827)	111 (9.86%)	Reference	Reference
Q2(1827 to < 4872)	84 (7.45%)	0.71 (0.49, 1.03) 0.074	0.67 (0.46, 0.97) 0.036
Q3(4872 to < 11,288)	75 (6.65%)	0.64 (0.44, 0.94) 0.022	0.58 (0.40, 0.86) 0.006
Q4(≥ 11,288)	68 (6.03%)	0.61 (0.42, 0.90) 0.013	0.59 (0.40, 0.87) 0.008
*P for trend*		0.009	0.005

^1^Values are ORs (95% CIs) unless otherwise indicated. CKD, Chronic kidney disease; DCI,dietary carotenoids intakes

model 1 was adjusted for age, race, BMI, smoke, FPG, TC, HDL, SUA.

model 2 was adjusted for age, race, BMI, smoke, FPG, TC, HDL, SUA, lipoprotein-lowering drugs, glucose-lowering drugs, diabetes, hypertension

### Subgroup analyses

**Fig. 2:**
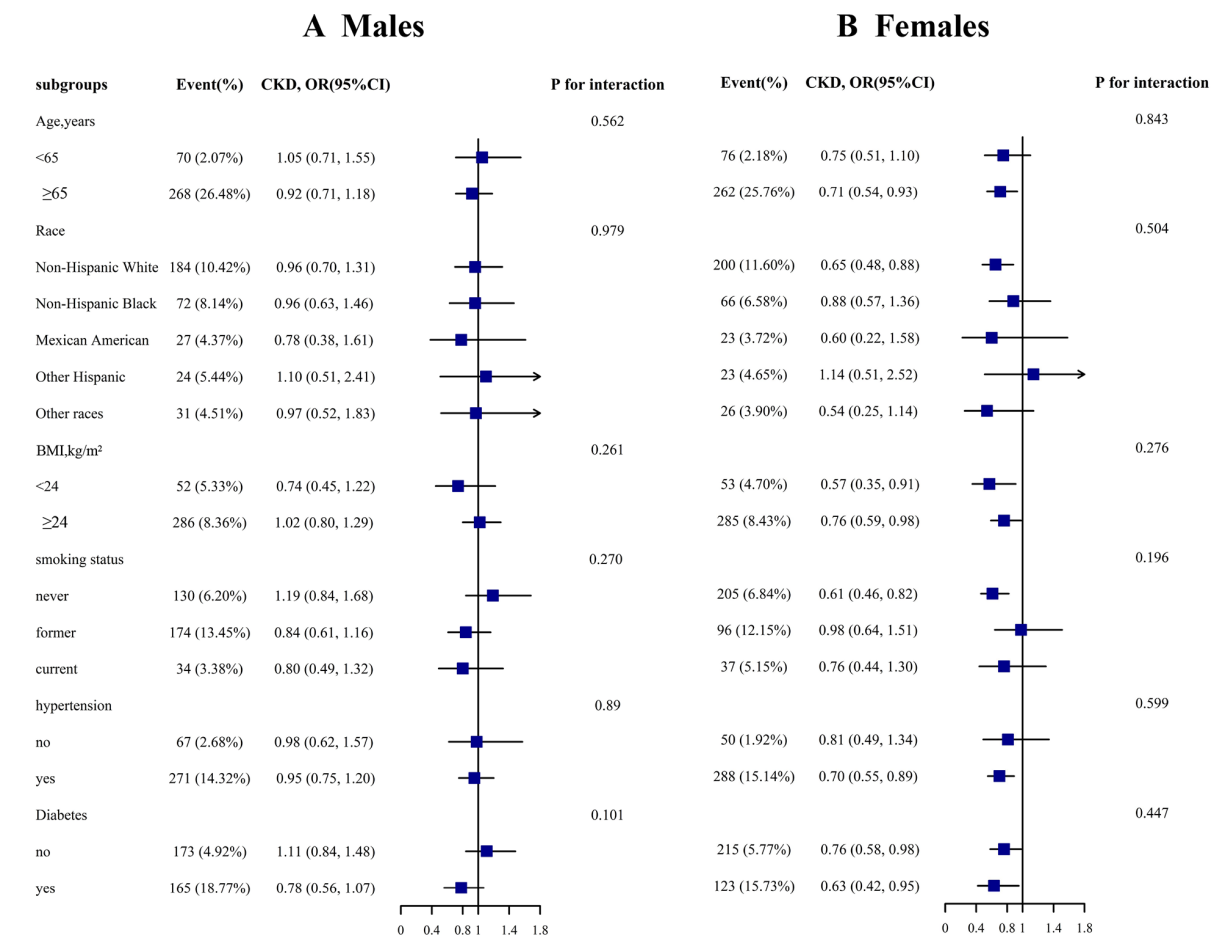
Stratified Analyses by Potential Modifiers of the Association between DCI and the prevalence of CKD by sex ^*^(A) males **(B)** females. *Each subgroup analysis adjusted for age, race, BMI, smoke, FPG, TC, HDL, SUA, lipoprotein-lowering, drugs, glucose-lowering drugs, diabetes, hypertension, except for the stratifying variable

We performed a subgroup analysis of the relationship between DCI and CKD in men and women (Fig. 2A and B). The relationship between DCI and CKD was not significantly affected in either men or women by the following factors: age (< 65 vs. ≥ 65 years), race (non-Hispanic White vs. non-Hispanic Black vs. Mexican American vs. other Hispanic vs. other races), BMI (< 24 vs. ≥24 kg/m^2^), smoking status (never vs. former vs. current), diabetes (no vs. yes), and hypertension (no vs. yes)(all P for interaction > 0.05).

## Discussion

There has been minimal research on the relationship between diet and risk of CKD. In this large cross-sectional study, increases in DCI were negatively correlated with CKD in females but not in males, and this association was independent of other risk factors for CKD.

To date, only Hirahatake et al. have carried out a comparable study. They used data from the Coronary Artery Risk Development in Young Adults (CARDIA) study to assess the relationship between serum carotenoids and rapid renal decline [18]. After 5 years of follow-up, 290 of the 2152 participants with baseline eGFR > 60 had rapid renal decline. Individuals in the highest quartile of serum carotenoids had significantly lower odds of a rapid decline in renal function (OR, 0.51; 95% CI, 0.32–0.80) in the fully adjusted model relative to individuals in the lowest quartile. In addition, animal studies support the protective effect of beta-carotenoids feeding on kidney of bromobenzene-treated rats [29, 30]. Evidence shows that higher intake of carotenoids improves the survival and prognosis of CKD patients and reduces the risk of all-cause death by 15% (HR = 0.85, 95% CI, 0.75–0.97, *P* = 0.011) [31].

We observed a significant correlation between the increase in DCI and a reduction in CKD prevalence among women, whereas no such association was found among men. Animal experiments have demonstrated comparable findings. Female mice exhibit higher bioavailability and conversion rates of carotenoids compared to male mice, as evidenced by a significant increase in plasma retinol levels [32–35]. Therefore, we hypothesize that the elevated bioavailability of carotenoids in female participants may result in increased plasma carotenoid levels, thereby enhancing their anti-inflammatory and antioxidant effects within the human body. Consequently, their protective effect on kidney function is likely to be more pronounced when compared to male participants. The number of male subjects who smoked in this study was 1294 (29.4%) and the number of subjects with diabetes was 879 (20.0%), with baseline SBP, DBP, and FPG values well above those of females. Compared with women, men had more risk factors for CKD, so the antioxidant effects of carotenoids in men were offset by the related risk factors, suggesting that we should pay more attention to the main risk factors such as blood glucose and blood pressure values of men. Moreover, preclinical and experimental studies show that systemic and vascular oxidative stress in men is higher than that in women [36–38]. These differences may be partly explained by the different expressions and/or activities of antioxidant enzymes such as superoxide dismutase, glutathione peroxidase, and NADPH oxidase) [39].

The mechanism by which DCI is associated with reduced risk of CKD is not clear. The alleviation of oxidative stress and inflammatory response may serve as a possible explanation. Oxidative stress is a part of the pathogenesis of CKD [40, 41]. In vitro and in vivo studies have shown that carotenoids are the most abundant fat-soluble phytochemicals in fruits and vegetables with antioxidant, anti-apoptotic and anti-inflammatory properties, many of which are related to the regulation of inflammatory and oxidative stress signaling pathways [42]. Carotenoids can eliminate reactive oxygen species (ROS) and enhance the cell’s ability to prevent oxidative stress [43].

### Limitations

Our study has several limitations: First, our research was based on NHANES data. This study is cross-sectional and does not allow for the establishment of a time correlation and causal inferences. Second, the dietary carotenoid intake was collected through two 24-hour recalls, which may lead to error due to memory bias and cannot accurately reflect the individual’s daily carotenoid intake. However, some studies have shown that two 24-hour recalls of daily dietary intake may be sufficient for evaluation [44]. Finally, although we adjusted for a wide range of potential confounders, the potential effects of unmeasured or residual confounders cannot be ruled out.

### Perspectives and significance

To sum up, our study provides evidence that carotenoid intake may prevent CKD in a gender-specific manner. In clinical practice, female patients can be encouraged to eat more foods rich in carotenoids to prevent the occurrence and development of CKD and related cardiovascular diseases, while for men, it is still the main risk factor for the control of CKD. Further studies are needed to understand the mechanistic basis of gender differences in the occurrence and development of CKD. Further research is needed to explain the role of sex.

## Conclusions

We demonstrated that higher DCI is associated with a decrease in CKD only in women, independent of other possible risk factors for CKD.

### Practical application

In clinical practice, we should recommend patients’ daily diet according to gender to prevent CKD and related complications.

## Data Availability

Publicly available datasets were analyzed in this study. This data can be found here: https://www.cdc.gov/nchs/nhanes/index.htm.
